# Analysis of the screening and predicting characteristics of the house-tree-person drawing test for mental disorders: A systematic review and meta-analysis

**DOI:** 10.3389/fpsyt.2022.1041770

**Published:** 2023-01-04

**Authors:** Huibing Guo, Bin Feng, Yingqiao Ma, Xueyi Zhang, Huiyong Fan, Zaiquan Dong, Taolin Chen, Qiyong Gong

**Affiliations:** ^1^Huaxi MR Research Center (HMRRC), Department of Radiology, West China Hospital of Sichuan University, Chengdu, Sichuan, China; ^2^Department of Student Affairs Management, West China School of Medicine, West China Hospital, Sichuan University, Chengdu, China; ^3^West China School of Medicine, Sichuan University, Chengdu, China; ^4^Research Unit of Psychoradiology, Chinese Academy of Medical Sciences, Chengdu, Sichuan, China; ^5^College of Medical Technology, West China Hospital of Sichuan University, Chengdu, China; ^6^Institute of Education, Bohai University, Jinzhou, China; ^7^Mental Health Center, West China Hospital of Sichuan University, Chengdu, China; ^8^Functional and Molecular Imaging Key Laboratory of Sichuan Province, West China Hospital of Sichuan University, Chengdu, Sichuan, China; ^9^Department of Radiology, West China Xiamen Hospital of Sichuan University, Xiamen, Fujian, China

**Keywords:** house-tree-person drawing test, mental disorders, screening, aiding diagnosis, meta-analysis

## Abstract

**Background:**

The house-tree-person (HTP) drawing test has received growing attention from researchers as a common projective test. However, the methods used to select and interpret drawing indicators still lack uniformity.

**Objective:**

This study aims to integrate drawing indicators into the process of screening for or classifying mental disorders by conducting a systematic review and meta-analysis of the application of the HTP test.

**Methods:**

A search of the following electronic databases was performed in May 2022: PubMed, Web of Science, Embase, EBSCO, CNKI, VIP, and Wanfang. Screening and checking of the literature were performed independently by two researchers. The empirical studies published on the use of the HTP test in mental disorders and studies providing specific data on the occurrence frequency of drawing characteristics were analyzed. A total of 30 studies were included in the meta-analysis, including 665 independent effect sizes and 6,295 participants. The strength of the association between drawing characteristics of the HTP test and the prevalence of mental disorders was measured by the ratio (OR) with a *95% CI*. Publication bias was assessed using a funnel plot, Rosenthal’s fail-safe number (*N*_fs_), and the trim and fill method.

**Results:**

The results revealed 50 drawing characteristics that appeared at least three times in previous studies, of which 39 were able to significantly predict mental disorders. The HTP test can be divided into the following four dimensions: house, tree, person, and the whole. These dimensions reflect the structure, size, and other characteristics of the picture. The results showed that the greatest predictor of mental disorders was the whole (OR = 4.20, *p* < 0.001), followed by the house (OR = 3.95, *p* < 0.001), the tree (OR = 2.70, *p* < 0.001), and the person (OR = 2.16, *p* < 0.001). The valid predictors can be categorized into the following four types: item absence, bizarre or twisted, excessive details, and small or simplified. The subgroup analysis showed that the affective-specific indicators included *no motion, leaning house, and decorated roof;* thought-specific indicators included *excessive separation among items*, *no window*, *loss of facial features*, and *inappropriate body proportions*; and common indicators of mental disorders included *no additional decoration*, *simplified drawing*, *very small house*, *two-dimensional house*, and *very small tree*.

**Conclusion:**

These findings can promote the standardization of the HTP test and provide a theoretical reference for the screening and clinical diagnosis of mental disorders.

## Introduction

Mental disorders are usually associated with distress or cognitive function, emotional regulation, or behavioral impairment. The prevalence of mental disorders has been increasing annually in recent years and has become a major contributor to the global disease burden (1, 2). One in every 8, or 970 million people in the world, lives with a mental disorder (3). There are many different types of mental disorders, which can be classified into thought-type disorders and affective-type disorders according to the main symptoms. Affective-type disorders include depression and anxiety (4), while thought-type disorders mainly include schizophrenia, paranoia, rumination, etc. (5).

Accurate screening and diagnosis should be performed before treating mental disorders to reduce their prevalence. However, current assessments mainly rely on scales, and these traditional measures have many drawbacks (6, 7). For example, it is difficult to assess the symptoms of patients with unclear self-perceptions based on scale questions. Moreover, due to social desirability, subjects may deliberately choose positive answers to hide their symptoms, resulting in a lack of valid results (8, 9).

As one of the three major testing techniques in psychology, projective testing can be used to compensate for the shortcomings of scales (10). The comprehensive use of various testing techniques is a future trend and can aid in the development of a projective test with better validity (11, 12). The house-tree-person (HTP) drawing test was proposed by Buck in 1948 and is currently one of the most widely used projective tests (13). According to a survey of 102 commonly used psychological tests conducted by the American Psychological Association, HTP ranks 8th in usage (14). The HTP test has the following advantages: initiative, structure, and non-verbal. On the one hand, it can conceal the test purpose and overcome the defensive psychology of subjects. On the other hand, painting is not affected by a subject’s culture and expression and thus can more accurately reflect personality traits and potential psychological problems (11, 15).

Many studies have applied the HTP test in screening and aiding the diagnosis of mental disorders. For example, one of the earliest studies examined whether the drawing characteristics in the HTP test were related to mental disorders, and they found a significant correlation between “line strength” and EEG; thus, line strength was thought to be a predictor of psychopathology (16). In addition, a psychological survey of 1906 college freshmen showed that the combined usage of HTP and SCL-90 increased the accuracy of screening for mental problems (17). HTP has also been found to be an effective tool for classifying personality disorders, depression, anxiety, and other mental disorders (18–20).

However, there are some shortcomings in the existing studies. One is that the scoring and interpretation of the HTP test are not standardized and lack consistency. The drawing characteristics selected by researchers are subjective, which makes it difficult to compare the results of different studies (21, 22). Moreover, the interpretations of some drawing characteristics are inconsistent. For instance, some researchers believe that drawing a “chimney” is a negative expression of family or internal conflict (23), while others believe that it is a positive characteristic indicating open communication channels with the outside world and the seeking of support and warmth (15, 24).

Although the above issues have received extensive attention from researchers, most of them have been presented in systematic reviews or research prospects (25–27). It is difficult to solve the problem through a theoretical summary alone. Therefore, we will integrate the drawing indicators of the HTP test of mental disorders through meta-analysis. Specifically, this study will answer the following three questions: (1) Which drawing characteristics have been frequently selected as screening indicators for mental disorders in previous studies? (2) How well do these drawing characteristics predict mental disorders? (3) Are there any differences in the predictive effects of the same drawing characteristics for affective-type disorders and thought-type disorders?

## Materials and methods

This study was conducted according to the Preferred Reporting Items for a Systematic Review and Meta-analysis (PRISMA) statement (28).

### Search strategy

To obtain studies to be used in the analysis, four English (PubMed, Web of Science, Embase, and EBSCO) and three Chinese (China National Knowledge Infrastructure (CNKI), Chinese Scientific Journal Database (VIP), and Wanfang) databases were utilized. The core search terms were “house-tree-person,” “HTP test,” “S-HTP,” “K-HTP,” “projective test,” and “drawing test.” The search period was initially from 1 January 1948 to 20 May 2022. Further details of the search strategy are displayed in the Supplementary material.

### Inclusion and exclusion criteria

For the retrieved literature, the inclusion or exclusion of meta-analysis was further judged according to the following criteria: (1) published empirical studies of the HTP test of mental disorders, excluding purely theoretical and literature review articles, were included; (2) the included studies distinguished between subjects with and without mental disorders using recognized and credible scales; (3) the included studies contrasted participants with mental disorders with those without mental disorders, and studies that focused only on those who had mental disorders were excluded; (4) the included studies provided specific data on the occurrence frequency of drawing characteristics, and studies where the original data were calculated in other forms or where the effect sizes could not be converted were excluded; and (5) all duplicate publications were excluded.

**FIGURE 1:**
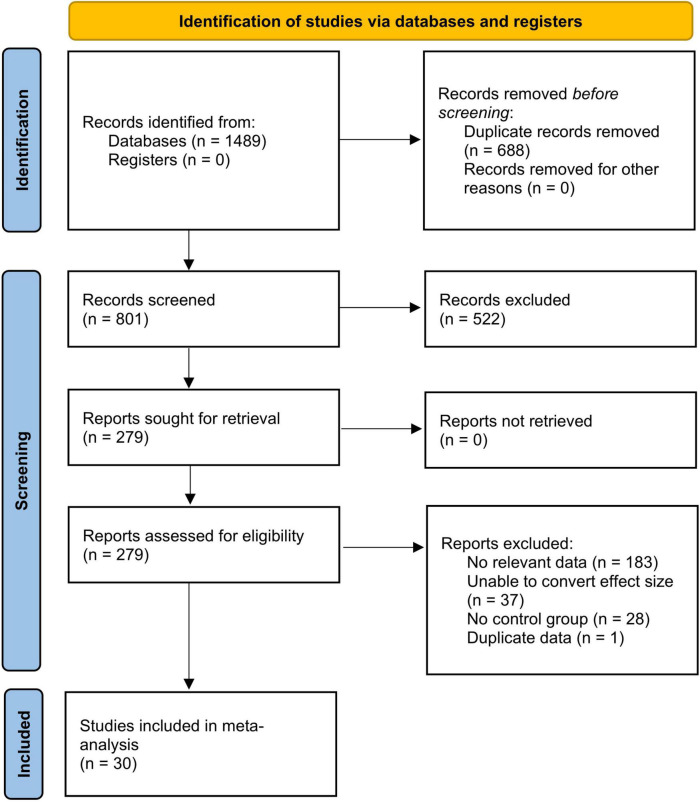
Flow chart of the identified studies.

Screening and checking of the literature were performed independently by two investigators (HBG and BF) based on the inclusion and exclusion criteria, and the consensus was negotiated in case of discrepancies. The final review was conducted by the corresponding author (TLC). A total of 1,498 potentially relevant studies were identified in different databases through the search strategy, with an initial screening achieved by scanning the titles and abstracts, followed by a full-text screening, resulting in the inclusion of 30 studies. The literature search and screening process is shown in Figure 1.

### Quality assessment

Quality was assessed using the Cross-Sectional Study Quality Assessment Forms (CSSQAF) recommended by the Agency for Healthcare Research and Quality. The form has 11 items, which receive a score of 0 if the result is “no” or “unclear” and 1 if the answer is “yes.” Publications with scores of 8-11, 4-7, and 0-3 were considered high-quality literature, moderate-quality literature, and low-quality literature, respectively. Two investigators (HBG and BF) independently rated the included literature and calculated the rater agreement coefficient, which was found to be good, with a Kappa value of 0.85.

### Coding procedures

As various studies used different names to describe the drawing characteristics, we standardized and unified the names. Three different naming principles were adopted according to the following cases: (1) when the same meaning but different wording was used, for example, *the house, tree, and person are clearly spaced*, and *excessive separation among items*; we adopted the expressions more frequently used by the predecessors; (2) when the meaning was the same but different directions were used, for example, *roots* and *no roots of trees*, the expressions more often by the predecessors were retained, and the opposite characteristics were scored in reverse; (3) when the meanings were similar but different wording was used, for example, *paintings without additional objects*, *no flowers and grass*, *painting without clouds*, as summary expression was utilized, such as *no additional decoration*. It should be noted that such characteristics should be carefully categorized. This process was carried out independently by two researchers (HBG and BF), and after completion, the agreement was reached after deliberation and discussion. In case of dispute, it was consulted by a third researcher (HYF) to resolve the issue.

In addition, the translation procedures used to translate Chinese drawing characteristics were as follows. First, two researchers (HBG and BF) independently translated the drawing characteristics into English, then discussed differences and merged them into version 1. Second, one researcher (ZQD) modified the grammar as well as the words and formed version 2. Third, back-translation was performed by two other researchers (HYF and YQM) and the translation was modified accordingly to ensure accuracy. Finally, the final version was formed by considering the three previous coding principles and maintaining consistency with English characteristic names. After completion, the corresponding author (TLC) reviewed it. Discussions and revisions continued if there was disagreement until all researchers reached a consensus.

### Statistical analysis

The strength of the association between drawing characteristics of the HTP test and the prevalence of mental disorders was measured by the ratio (OR) with a *95% CI*. Significance was determined by the *Z*-test, and *p* < 0.05 was considered statistically significant. The included literature was tested for heterogeneity and evaluated comprehensively using the Q test and the *I*^2^ statistic according to the Cochrane Handbook for Systematic Reviews of Interventions. The Q test obeys a Chi-square test distribution, and when Q ≤ 0.10, the heterogeneity test is considered statistically significant, while *I*^2^ reflects the proportion of between-study variation attributable to heterogeneity, rather than random error or chance. *I*^2^ values of 25, 50, and 75% represent low, moderate, and high heterogeneity, respectively, and a random effects model is more appropriate when heterogeneity is high (29).

### Publication bias

Publication bias was assessed using a funnel plot, Rosenthal’s fail-safe number (*N*_fs_), and the trim and fill method. If the effect values were concentrated at the top of the funnel plot and clustered roughly symmetrically around the mean, there was no publication bias. In addition, the larger the fail-safe number is, the less likely bias is, which means that it is less likely that the conclusions will be overturned. If *N*_fs_ < 5k + 10 (k is the original number of studies), publication bias should be considered (30). The trim and fill methods distribute the studies as symmetrically as possible on the left and right sides of the mean effect size by first cutting and then complementing and re-estimating the true value of the combined effect size (31). If the effect size does not change significantly after cutting and complementing, then publication bias can be considered not to exist. All statistics for this meta-analysis were calculated by CMA 3.0 software.

## Results

### Study selection and characteristics

A total of 30 studies were included in this meta-analysis, all of which were cross-sectional studies, including 10 in English and 20 in Chinese. A total of 665 independent effect sizes were included, and 6,295 subjects participated in the survey. The results of the quality assessment showed that 19 of the publication included in this study scored between 7 and 9, which indicates high quality, and 11 scored between 4 and 6, which indicates moderate quality. For the studies included in the meta-analysis, the following information was extracted: (1) first author and time of publication; (2) version of the HTP test used for the study; (3) total sample size, including the number of subjects in the mental disorder and control groups; and (4) the type of mental disorder and screening tool. The results are shown in Table 1.

**TABLE 1:** Studies included in the meta-analysis.

Author	Year	HTP type	Sample size	Disease group	Control group	Mental disorders	Scales	Score
Chen	2015	S-HTP	60	30	30	Schizophrenia	SCL-90, BPRS	8
Chen	2015	S-HTP	562	38	524	Dependent personality disorder	PDQ-4+	7
Deng	2014	S-HTP	64	32	32	Schizophrenia	BPRS	8
Deng	2017	S-HTP	60	30	30	Depression	–	4
Eisel	1978	HTP	138	69	69	Schizophrenia	DSM	8
Fukunishi	2002	S-HTP	192	50	142	Alexithymia	TAS-20	7
Kirchner	1974	HTP	195	49	146	Substance addiction disorder	–	4
Koide	1992	HTP	126	16	110	Organic mental disorders	–	5
Kwark	2017	S-HTP	100	50	50	Schizophrenia	–	5
Lee	2019	S-HTP	186	23	163	Depression	EPQ, PHQ-9	6
Lee	2020	S-HTP	186	60	126	Substance addiction disorder	NDSS	6
Li	2014	S-HTP	105	35	70	High-functioning-autism	DSM	8
Li	2016	S-HTP	65	30	35	Depression	HAMD	4
Li	2020	S-HTP	324	190	134	Anxiety	MSSMHS	9
Li	2021	S-HTP	60	30	30	Depression	SCL-90	5
Ning	2015	S-HTP	676	148	528	Depression	CDI	8
Sheng	2019	S-HTP	167	27	140	Anxiety	SAS	6
Wang	2007	S-HTP	55	25	30	Mental disease	SCL-90	7
Wang	2017	S-HTP	177	74	103	Anxiety	MHT	6
Wang	2019	S-HTP	71	-	-	Anxiety, depression, paranoia	SCL-90	8
Xiang	2020a	HTP	358	22	336	Attention deficit/hyperactivity disorder	CBCL	7
Xiang	2020b	HTP	358	68	290	Depression	CBCL	7
Xie	1994	S-HTP	220	110	110	Schizophrenia	–	5
Yan	2014	S-HTP	540	277	263	Depression	SDS	8
Yang	2019	S-HTP	167	57	110	Depression	SDS	9
Zhao	2015	HTP	170	37	133	Somatization disorder	CSI, CBCL	8
Zhou	2019	S-HTP	39	17	22	Schizophrenia	SAPS, SANS	7
Zhou	2021	S-HTP	200	100	100	Rumination	RRS	9
Zhu	2011	S-HTP	112	59	53	Post-traumatic stress disorder	PCL-C	8
Zhu	2020	S-HTP	562	140	422	Narcissistic personality disorder	PDQ-4+	7

SCL-90, symptom checklist 90; BPRS, brief psychiatric rating scale; PHQ-9, patient health questionnaire-9 items; DSM, diagnostic and statistical manual of mental disorders; TAS-20, Toronto Alexithymia scale; EPQ, Eysenck personality questionnaire; NDSS, nicotine dependence syndrome scale; HAMD, Hamilton depression scale; MSSMHS, middle school student mental health scale; CDI, children’s depression inventory; SAS, self-rating anxiety scale; MHT, mental health test; CBCL, Achenbach Child Behavior Checklist; SDS, self-rating depression scale; CSI, children’s somatization inventory; SAPS, scale for assessment of positive symptoms; SANS, scale for assessment of negative symptoms; RRS, ruminative responses scale; PCL-C, PTSD checklist-civilian version; PDQ-4+, personality diagnostic questionnaire-4+.

### Predictive effect of mental disorders

Of the 30 included studies, 341 different drawing characteristics of the HTP drawing test were found; of which, 5 appeared more than or equal to 10 times, 20 appeared 5 to 10 times, 25 appeared 3 to 5 times, and 289 appeared 1 to 3 times. A total of 50 drawing characteristics with a high frequency (more than or equal to 3 times) were selected for inclusion in the analysis to explore their validity in screening for mental disorders (32). The HTP test can be divided into the following four dimensions that reflect the structure, size, and other characteristics of the picture: house, tree, person, and the whole drawing. The predictive effects of the four dimensions regarding mental disorders were in the following order: the whole drawing (OR = 4.20, *p* < 0.001) had the highest effect, followed by the house drawing (OR = 3.95, *p* < 0.001), the tree drawing (OR = 2.70, *p* < 0.001), and finally, the person drawing (OR = 2.16, *p* < 0.001). The predictive effects of specific drawing characteristics for each dimension are shown in Table 2.

**TABLE 2:** The predictive effect of drawing characteristics on mental disorders.

	Drawing characteristics	*k*	Heterogeneity		OR	95% CI	*P*	*N*_fs_
			*Q(p)*	*I^2^(%)*				
Whole	No additional decoration	15	0.000	93.70	2.59	1.25∼10.29	0.041	197
12 items	Excessive separation among items	10	0.000	88.36	3.84	1.95∼7.56	0.000	199
	Simplified drawing	6	0.001	76.15	9.64	4.08∼22.75	0.000	170
	Weak or intermittent lines	5	0.196	33.79	3.19	2.03∼5.01	0.000	35
	No motion	5	0.000	85.41	2.96	1.46∼6.00	0.003	63
	Omitted house, tree or person	5	1.174	33.61	2.81	1.52∼5.18	0.001	14
	Small drawing size	4	0.182	38.32	5.71	3.37∼9.68	0.000	43
	Shaded or blackened drawing	4	0.013	71.99	2.72	1.60∼4.62	0.000	17
	Scribbled drawing	4	0.117	49.12	2.56	1.52∼4.32	0.000	13
	Emphasis on straight lines	3	0.000	88.19	11.75	2.16∼64.02	0.004	41
	No theme	3	0.805	0.00	9.36	3.74∼23.4	0.000	14
	Shadow	3	0.411	0.00	2.88	1.36∼6.11	0.006	5
	Total dimensional score	–	0.000	88.47	4.20	3.06∼5.77	0.000	–
House	Very small house	7	0.000	88.88	4.24	1.91∼9.44	0.000	193
9 items	No door	6	0.035	58.25	4.52	2.96∼6.92	0.000	71
	No window	6	0.001	74.89	3.09	2.02∼4.72	0.000	51
	Decorated roof	6	0.000	75.25	2.32	1.27∼4.25	0.006	65
	Leaning house	5	0.002	76.32	2.68	1.49∼4.81	0.001	52
	Two-dimensional house	5	0.003	74.48	1.76	1.38∼2.24	0.000	23
	Smoking chimney	4	0.078	55.96	2.27	1.43∼3.61	0.001	14
	Shaded or blackened wall	4	0.014	71.57	1.66	1.01∼2.71	0.044	0
	Bizarre house	3	0.092	58.12	4.64	2.56∼8.40	0.000	19
	Total dimensional score	–	0.000	76.94	3.09	2.42∼3.95	0.000	–
Tree	Very small tree	11	0.000	82.91	2.65	1.41∼4.97	0.002	123
7 items	Roots	6	0.139	39.93	4.35	2.96∼6.39	0.000	70
	Truncated tree	6	0.003	72.67	2.90	1.62∼5.18	0.000	24
	Sharp branch	6	0.007	68.60	2.35	1.60∼3.46	0.000	6
	Bizarre tree	4	0.025	67.94	2.78	1.91∼4.04	0.000	31
	Dead tree	4	0.362	6.14	2.67	1.59∼4.47	0.000	14
	Flattened crown	3	0.038	69.54	2.82	1.91∼4.17	0.000	13
	Total dimensional score	–	0.000	69.76	2.70	2.34∼3.11	0.000	–
Person	Loss of facial features	10	0.000	82.86	2.71	1.46∼5.04	0.002	94
11 items	Shaded or blackened person	5	0.002	76.79	4.63	1.45∼14.85	0.010	27
	Poker face	5	0.150	40.65	2.09	1.40∼3.12	0.000	16
	Inappropriate body proportions	5	0.006	72.10	1.99	1.37∼2.88	0.000	21
	Single line limbs	5	0.117	45.77	1.93	1.32∼2.81	0.001	18
	Negative expression	4	0.300	18.15	3.59	1.96∼6.59	0.000	8
	Bizarre person	4	0.105	51.16	3.18	1.49∼6.77	0.003	6
	Complete or partial loss of limbs	4	0.084	54.92	1.82	1.26∼2.63	0.001	6
	Incomplete person	3	0.139	49.30	4.90	3.05∼7.88	0.000	30
	Very small person	3	0.094	57.64	3.02	2.04∼4.45	0.000	25
	Fist	3	0.972	0.00	3.66	1.70∼7.85	0.001	6
	Total dimensional score	–	0.000	66.53	2.16	2.22∼3.49	0.000	–

### Whole drawing characteristics

Of the 15 whole drawing characteristics, 12 were significant predictors of mental disorders. The significant characteristics in order of OR size were the *emphasis on straight lines* (OR = 11.75, *p* = 0.004), *simplified drawing* (OR = 9.64, *p* < 0.001), *no theme* (OR = 9.36, *p* < 0.001), s*mall drawing size* (OR = 5.71, *p* < 0.001), *excessive separation among items* (OR = 3.84, *p* < 0.001), *weak or intermittent lines* (OR = 3.19, *p* < 0.001), *no motion* (OR = 2.96, *p* = 0.003), *shadow* (OR = 2.88, *p* = 0.006), *omitted house, tree or person* (OR = 2.81, *p* = 0.001), *shaded or blackened drawing* (OR = 2.72, *p* < 0.001), *no additional decoration* (OR = 2.59, *p* = 0.041), and *scribbled drawing* (OR = 2.56, *p* < 0.001). In contrast, characteristics such as *emphasizing the horizon*, *weighted or repeated lines*, and the *fence* did not significantly predict mental disorders (*p* > 0.05).

### House drawing characteristics

Of note, 9 of the 12 house drawing characteristics that significantly predicted mental disorders were ranked according to OR: *bizarre house* (OR = 4.64, *p* < 0.01), *no door* (OR = 4.52, *p* < 0.001), *very small house* (OR = 4.24, *p* < 0.001), *no window* (OR = 43.09, *p* < 0.01), *leaning house* (OR = 2.68, *p* = 0.01), *decorated roof* (OR = 2.32, *p* = 0.006), *smoking chimney* (OR = 2.27, *p* = 0.001), *two-dimensional house* (OR = 1.76, *p* < 0.001), and *shaded or blackened wall* (OR = 1.66, *p* = 0.044). However, *chimney* and *closed door or window* were not statistically significant in predicting mental disorders (*p* > 0.05).

### Tree drawing characteristics

Of the 9 tree drawing characteristics, 7 were significant predictors for mental disorders. The significant characteristics in order of OR were as follows: *roots* (OR = 4.35, *p* < 0.001), *truncated tree* (OR = 2.90, *p* < 0.001), *flattened crown* (OR = 2.82, *p* < 0.001), *bizarre tree* (OR = 2.78, *p* < 0.001), *dead tree* (OR = 2.67, *p* < 0.001), *very small tree* (OR = 2.65, *p* = 0.002), and *sharp branch* (OR = 2.35, *p* < 0.001). In contrast, *scars of trees* and *shaded or blackened trees* were not significant predictors of mental disorders (*p* > 0.05).

### Person drawing characteristics

Notably, 11 of the 14 person drawing characteristics were significant predictors for mental disorder, ranked according to OR as follows: *incomplete person* (OR = 4.90, *p* < 0.001), *shaded or blackened person* (OR = 4.63, *p* = 0.010), *fist* (OR = 3.66, *p* = 0.001), *negative expression* (OR = 3.59, *p* < 0.001), *bizarre person* (OR = 3.18, *p* = 0.003), *very small person* (OR = 3.02, *p* < 0.001), *loss of facial features* (OR = 2.71, *p* = 0.002), *poker face* (OR = 2.09, *p* < 0.001), *inappropriate body proportions* (OR = 1.99, *p* < 0.001), *single line limbs* (OR = 1.93, *p* = 0.001), and *complete or partial loss of limbs* (OR = 1.82, *p* = 0.001). However, *simple person*, *fingers*, and *not frontal portrait* did not significantly predict mental disorders (*p* > 0.05).

### Subgroup analysis

As Table 2 shows, there was very high heterogeneity (*I^2^* > 75%) in 11 drawing characteristics, and 6 characteristics with *I*^2^ values between 70 and 75% also had high heterogeneity. We conducted a subgroup analysis of the above characteristics. The results are shown in Table 3.

**TABLE 3:** Subgroup analysis of mental disorder types.

	Drawing characteristics	Type	*k*	Heterogeneity		OR	95% CI	*P*
				*Q(p)*	*I^2^(%)*			
ASI	No motion	AD	3	0.000	86.99	3.34	1.22∼9.16	0.019
		TD	2	0.001	91.06	2.63	0.63∼11.02	0.185
	Leaning house	AD	2	0.082	66.96	2.13	1.48∼3.07	0.000
		TD	2	0.002	89.06	3.77	0.87∼16.43	0.077
	Decorated roof	AD	3	0.000	85.16	2.49	1.50∼4.13	0.000
		TD	2	0.179	44.63	2.00	0.70∼5.77	0.197
TSI	Excessive separation among items	AD	4	0.000	92.49	2.47	0.79∼7.71	0.119
		TD	4	0.000	87.21	6.09	1.40∼26.41	0.016
	No window	AD	2	0.019	81.68	3.14	0.24∼41.20	0.385
		TD	3	0.928	0.00	6.41	3.53∼11.65	0.000
	Loss of facial features	AD	5	0.000	88.61	1.81	0.75∼4.35	0.185
		TD	3	0.025	72.82	2.60	1.62∼4.16	0.000
	Inappropriate body proportions	AD	2	0.865	0.00	1.29	0.71∼2.33	0.398
		TD	2	0.473	0.00	9.29	3.77∼22.90	0.000
MDC	No additional decoration	AD	6	0.000	94.23	1.38	0.45∼4.19	0.000
		TD	5	0.004	74.40	14.19	8.72∼23.08	0.000
	Simplified drawing	AD	2	0.000	94.60	13.06	1.12∼152.54	0.041
		TD	3	0.424	0.00	7.23	3.66∼14.30	0.000
	Very small house	AD	3	0.000	95.75	5.29	1.16∼24.21	0.032
		TD	3	0.149	47.44	3.87	2.09∼7.16	0.000
	Two-dimensional house	AD	2	0.024	80.40	3.00	0.99∼9.14	0.050
		TD	2	0.257	22.14	2.08	1.30∼3.36	0.002
	Very small tree	AD	6	0.003	72.01	2.70	1.96∼3.72	0.000
		TD	4	0.128	47.27	3.92	2.34∼6.59	0.000

ASI, affect-specific indicators; TSI, thought-specific indicators; MDC, mental disorders coindicators; AD, affective-type disorders; TD, thought-type disorders.

In the subgroup analysis, mental disorders were classified into affective-type disorders (depression and anxiety) and thought-type disorders (schizophrenia, paranoia, and rumination). Drawing characteristics that appeared more than twice in both disorders (12 items in total) were extracted based on the number of studies after classification. The results showed that some drawing characteristics were significant predictors of affective-type disorders, but not of thought-type disorders, including *no motion* (OR = 3.34, *p* = 0.019), *leaning house* (OR = 2.13, *p* < 0.001), and *decorated roof* (OR = 2.49, *p* < 0.001), which could be known as affective-specific indicators. Conversely, drawing characteristics that significantly predicted thought-type disorders, but not affective-type disorders, included *excessive separation among items* (OR = 6.09, *p* = 0.016), *no window* (OR = 6.41, *p* < 0.001), *loss of facial features* (OR = 2.60, *p* < 0.001), and *inappropriate body proportions* (OR = 9.29, *p* < 0.001), which are thought-specific indicators. Furthermore, *no additional decoration*, *simplified drawing*, *very small house*, *two-dimensional house*, and *very small tree* were significant predictors of both mental disorders (*p* < 0.01) and could be described as mental disorder coindicators.

### Analysis of publication bias

**FIGURE 2:**
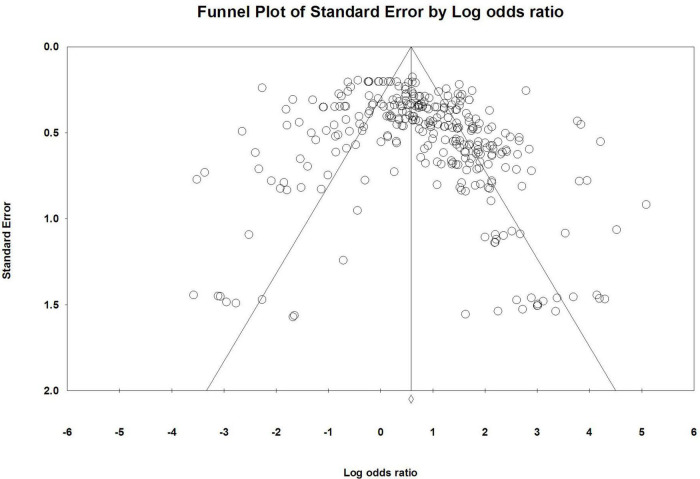
Funnel plot for the study of the relationship between drawing characteristics and mental disorders.

The funnel plot (Figure 2) showed that most of the effect sizes were located at the top of the funnel plot and were largely evenly clustered on either side of the mean effect values. It can be preliminarily judged that the possibility of publication bias in this meta-analysis is low. However, since the funnel plot evaluation was relatively subjective, the publication bias of each drawing characteristic was further judged according to the loss of safety factor (*N*_fs_), and the results are shown in Table 2.

There was no publication bias, and the conclusion was more reliable for the drawing characteristics with *N*_fs_ > 5k + 10. For the painting features that did not meet this criterion, the results were further examined by the trim and fill method and are shown in Table 4. All drawing characteristics showed significant effect sizes except for the *shaded or blackened person* characteristic, which can be considered not to have significant publication bias. The significance of this characteristic should be interpreted with caution, probably due to the small number of published studies or the small effect size.

**TABLE 4:** Analysis of publication bias.

	Drawing characteristics	Trim and fill imputed studies	OR	Adjusted OR	95% CI
Whole	Omitted house, tree or person	3	2.81	1.87	1.09∼3.20
	Shaded or blackened drawing	1	2.72	2.43	1.44∼4.08
	Scribbled drawing	2	2.56	1.71	1.08∼2.71
	No theme	1	9.36	8.62	3.59∼20.69
	Shadow	0	2.88	2.88	1.36∼6.11
House	Two-dimensional house	1	1.76	1.61	1.28∼2.04
	Smoking chimney	2	2.27	1.73	1.13∼2.64
	Shaded or blackened wall	0	1.66	1.66	1.01∼2.71
	Bizarre house	0	4.64	4.64	2.56∼8.40
Tree	Truncated tree	2	2.90	2.13	1.23∼3.71
	Sharp branch	1	2.35	2.18	1.50∼3.18
	Dead tree	2	2.67	2.36	1.44∼3.88
	Flattened crown	0	2.82	2.82	1.91∼4.17
Person	Shaded or blackened person	3	4.63	1.32	0.95∼1.83
	Poker face	2	2.09	1.65	1.14∼2.39
	Inappropriate body proportions	1	1.99	1.77	1.23∼2.54
	Single line limbs	2	1.93	1.73	1.20∼2.51
	Negative expression	1	3.59	3.34	1.97∼5.67
	Bizarre person	1	3.18	2.75	1.32∼5.72
	Complete or partial loss of limbs	0	1.82	1.82	1.26∼2.63
	Fist	0	3.66	3.66	1.70∼7.85

## Discussion

Projection theory suggests that drawing is an expression of the subconscious, and the size and other characteristics of the drawing reflect an individual’s emotions, personality, etc. (21). Many studies have demonstrated the use of the HTP drawing test to screen for mental disorders. However, there was a serious lack of consistency in the drawing characteristics selected in previous studies, which made it difficult to compare the different findings. Additionally, the predictive effects of some drawing characteristics were inconsistent. In this study, we found through meta-analysis that the predictive effects of the HTP test’s four dimensions on mental disorders were in the following order: the effect of the whole drawing was the greatest, followed by house drawing, tree drawing, and person drawing. Furthermore, we focused on integrating drawing characteristics that were used more frequently in previous studies and identified 39 significant predictors of mental disorders.

Psychodynamic theory suggests that behavior is driven or motivated by internal forces, with a focus on the unconscious, defense mechanisms, projections, etc. (33). Referring to this, HTP drawing characteristics can be categorized into the following four types: *Item absence*, *bizarre or twisted*, *excessive details*, and *small or simplified*. First, *item absence* reflects the loss of self-awareness, or strong psychological defenses, and can be thought of as an individual’s repression of self. Second, *bizarre or twisted* implies psychological conflict or a sense of unreality that inner and external environments are inconsistent. Third, *excessive details* suggest that internal conflicts have led to obvious anxiety, which manifests as nervousness, sensitivity, and irritability. Finally, *small or simplified* reflects avoidance and retreat due to low mental motivation and energy. In the following, we discuss the drawing characteristics of each type and summarize them in Table 5.

**TABLE 5:** Characteristics and implications of effective predictive drawing for mental disorders.

Type	Drawing characteristics	Indicates meaning
Item absence	Excessive separation among items, omitted house, tree or person, no door, no window, loss of facial features, poker face, complete or partial loss of limbs, and incomplete person	Loss of self-awareness and psychological defenses
Bizarre or distortion	Leaning house, bizarre house, truncated tree, dead tree, bizarre tree, sharp branch, flattened crown, bizarre person, inappropriate body proportions, and fist	Psychological conflict and sense of unreality
Excessive details	Shaded or blackened drawing, shadow, decorated roof, smoking chimney, shaded or blackened wall, roots, shaded or blackened person, and negative expression	Nervousness, sensitivity, and irritability
Small or simplified	No additional decoration, simplified drawing, no motion, no theme, small drawing size, weak or intermittent lines, emphasis on straight lines, scribbled drawing, very small house, two-dimensional house, very small tree, very small person, and single line limbs	Low mental motivation, avoidance and retreat

### Drawing characteristics of the house-tree-person test

#### Whole drawing characteristics

We found that the whole drawing characteristics were the best predictors of mental disorders, and the significant specific characteristics were as follows: (1) Item absence: this characteristic represents the absence of something in the picture that should be included, and two drawing characteristics are included, namely, *omitted house, tree, or person*, and *excessive separation among items.* The present study found that the absence of a house, tree, or person, or distance between them was a significant predictor of a mental disorder, which is consistent with the results of previous studies (23, 27). A drawing in which the house, tree, and person are complete and at appropriate distances reflects regularity and high personal reality satisfaction (34). In contrast, the absence of items from the whole drawing indicates strong defensiveness or lack of social support. (2) Excessive details: this characteristic indicates that some unnecessary characteristics have been drawn, including *shaded or blackened drawings* and *shadows*. Jung highlighted that shadows represent the hidden or unconscious psychology within the individual, and the presence of shadows and blackening indicates that the illustrator is autistic and or is experiencing (35). We found that the drawing of shadows or shading was a significant predictive feature of a mental disorder, which is consistent with many previous studies (15, 36). Thus, the inclusion of excessive details in the whole drawing is an important indicator of inner anxiety. (3) Small or simplified: in this whole drawing characteristic, the picture is drab and meaningless, and specific drawing characteristics include *no additional decoration*, *simplified drawing*, *no motion*, *no theme*, *small drawing size*, *weak or intermittent lines*, *emphasis on straight lines*, and *scribbled drawing.* Previous researchers have paid more attention to the decorations of the drawing. *No additional decoration* other than the house, tree, and person usually represents low psychological energy and a lack of enthusiasm and motivation in life. A study of schizophrenia supported this view and found a significant enrichment of drawings after the patients were treated (37). Both the *no motion* and *no theme* characteristics predict mental disorders. Previous studies have also found that the drawings of depressed patients are more likely to lack emotion and theme (38). In addition, the size of the picture is usually related to the self-awareness and psychological state of the painter. A *small drawing size* indicates that the subject may have a low self-evaluation or be insecure (19). Moreover, *weak or intermittent lines* often suggest indecision, as well as unclear self-awareness and emotional tendencies (39), and are more likely to be reflected in patients with mental disorders. Thus, a small or simplified whole drawing reflects low mental energy, avoidance, and withdrawal.

#### House drawing characteristics

By analyzing the size of the house, windows and doors, the floor, etc., the family atmosphere, self-image, and interpersonal status of the illustrator can be revealed (40). In this study, we found that the significant house drawing characteristics are as follows: (1) Item absence: this characteristic means that the house is missing necessary features, such as *no door* and *no window.* Doors and windows are channels of contact with the outside world, and *no door* suggests strong defensiveness (38), corresponding to the self-closure and refusal to communicate in patients with mental disorders. Both *no door* and *no window* were found to be significant predictive characteristics of mental disorders in this meta-analysis. Chen (37) also noted that there is a significant increase in doors and windows after schizophrenic patients receive treatment. Thus, the absence of items in the house drawing indicates an individual’s autism and defensiveness. (2) Bizarre or distortion: this characteristic means that the shape or features of the house deviate from reality, such as a *leaning house* or a *bizarre house*. *A leaning house* suggests unbearable stress and can significantly predict a mental disorder (23). Some researchers have found that *bizarre houses* (e.g., churches, temples, and pavilions) are also more likely to appear in the paintings of schizophrenic patients (41). Thus, bizarre houses or distortion of house drawing reflects inner repression and escape from reality. (3) Excessive details: this characteristic represents an excessive house depiction, including the following specific characteristics: *decorated roof*, *smoking chimney*, *shaded, or blackened walls*. Some researchers have argued that individuals with high activity levels usually create more meticulous drawings, and the opposite is true for individuals with low activity levels, such as those suffering from depression (42); however, others have suggested that detailed delineation represents neuroticism, sensitivity, and irritability (36, 38). The results of this meta-analysis supported the latter, showing that a *decorated roof* and *walls that are shaded or painted black* were both significant predictors of mental disorders. In addition, a *smoking chimney* indicates that the subject is experiencing family conflict, anxiety, and tension (37) and has a positive predictive effect regarding mental disorders. Therefore, excessive details of a house drawing reflect an individual’s concern for family and the apparent anxiety. (4) Small or simplified: this house drawing characteristic indicates that the house drawing is too simple or flat, including *very small houses* and *two-dimensional houses*. The house size usually represents the family relationship and status of the artist, and v*ery small houses* are mostly seen in families with low intimacy and prominent conflicts (37). Deng (39) found that 84.4% of the schizophrenia group painted houses that were too small, which was significantly higher than that of the normal group (34.4%). *A two-dimensional house* appears monotonous and lacks dimensionality, which usually reflects introverted and withdrawn personalities and is more likely to appear in the drawings of depressed individuals (43). Thus, a small or simplified house drawing reflects low security and poor intimacy.

#### Tree drawing characteristics

Many projective tests have used tree imagery as a theme; in addition to the HTP test, a common test using this theme is the tree test (44). Tree imagery often reflects emotional experiences related to growth and can reflect the relationship between an individual’s subjective feelings and the external environment (21). The results showed that the significant characteristics of a tree drawing include the following: (1) Bizarre or distortion: this tree imagery has characteristics that are different from usual, including *truncated trees*, *sharp branches*, *bizarre trees*, *dead* trees, and *flattened crowns*. *Truncated trees* or *dead trees* often symbolize emotional indifference, lack of vitality, and loss of willingness to live (45, 46) and can significantly predict mental disorders. Hui (38) also found that *dead trees* emerged only in the depressed group. In addition, a *flattened canopy* indicates that external stress overwhelms subjects (26), which is supported by the results of this meta-analysis. *Sharp branches* are often thought to be associated with aggression and destructiveness. Chen (37) found that the percentage of *sharp branches* drawn by schizophrenic patients decreased from 37.7 to 6.7% once they received treatment. Therefore, bizarre or distorted tree drawings mainly reflect the unrealistic and aggressive traits of individuals. (2) Excessive details: this characteristic implies complex depictions of tree characteristics, such as *roots*. *Roots* indicate an immature mind and internal conflict (39), and the results of the meta-analysis demonstrate that the trait is one of the indicators of mental disorders. (3) Small or simplified: this tree drawing characteristic means that the tree imagery is too simple, and the significant characteristic is a *very small tree*. Tree imagery symbolizes lives and energy. Large trees represent vitality, while *very small trees* imply loneliness and a lack of self-confidence, which are more likely to appear in the paintings of patients with mental disorders (46).

#### Person drawing characteristics

The person’s imagery often directly reflects the participant’s self-concept (40). In addition to the HTP test, the human drawing test is also widely used in clinical assessment (47). We found that multiple drawing characteristics of a person could predict mental disorders, including the following: (1) Item absence: this characteristic means that the figure is drawn with incomplete characteristics such as facial features or limbs, including *an incomplete person*, *loss of facial features*, *poker face*, *complete or partial loss of limbs*. Machover (48) indicated that an *incomplete person* represents an incomplete self-image. If a part of the figure is omitted from the painting, this signals the loss of function of that part. *Complete or partial loss of limbs* also reflects the loss of self-awareness and even the lack of will to live in patients with mental disorders (27). Therefore, the absence of items in the drawing of a person means that the individual’s self-awareness is weak or even lost. (2) Bizarre or distortion: this characteristic represents that the body is disproportionate or has uncommon features, such as *inappropriate body proportions*, *a bizarre person*, and the drawing of a *fist*. *A bizarre person* or *inappropriate body proportions* imply conflict between individuals and the external environment and are more likely to appear in the drawings of patients with mental disorders, consistent with many previous studies (49, 50). The drawing of a *fist* has a similar meaning to that of a sharp branch, indicating strong aggression and rebelliousness (13, 48), and is also a significant predictor of mental disorders. Thus, bizarre or distorted person’s drawings reflect the individual’s conflict or aggressiveness toward the external environment. (3) Excessive details: this characteristic represents that the figure drawing is depicted in unreasonable detail, such as a *shaded or blackened person* and *negative expression*. Researchers have argued that *shaded or blackened persons* imply the melancholy and depressed state of the painter (51), and the results of the meta-analysis support this view. In addition, *negative expressions* (e.g., sadness and anger) tend to reflect negative emotions and are more likely to be expressed in persons with mental disorders (52). Therefore, excessive details in the person’s drawing usually reflect an individual’s negative emotions, such as depression and anxiety. (4) Small or simplified: this person drawing characteristic indicates a person drawing that is too small or oversimplified, and includes the following two significant characteristics: *very small person* and *single line limbs*. Figure size is important for explaining individual self-awareness, and a *very small person* symbolizes weak self-awareness and low mental energy in subjects (53) and appears in a much higher proportion of patients with mental disorders than in normal groups (34). *Single line limbs* mean that the figure drawing is overly simple and abstract; this characteristic almost exclusively occurs in patients with psychiatric disorders and is a significant predictive feature of disorders such as schizophrenia (41, 54). Thus, a small or simplified person drawing reflects weak self-awareness and low self-esteem.

#### Subgroup analysis

Furthermore, we know that there are differences in clinical symptoms between affective-type disorders and thought-type disorders. According to projective theory, it can be speculated that the differences would be reflected in the drawing characteristics. Therefore, we further explored the independent predictive characteristics of these two mental disorders through heterogeneity analysis. The results support the hypothesis, showing that some characteristics can only predict a specific type of mental disorder, while some characteristics have the same predictive effect for both types of mental disorders. We present the affective-specific indicators, thought-specific indicators, and common indicators separately below. These findings could provide a more practical reference for the screening and diagnosis of different types of mental disorders.

Affective-specific indicators included *no motion, leaning house*, and *decorated roof*. *No motion* is an important reflection of emptiness, reflecting a depressed mood and lack of mental motivation, which coincides with the clinical manifestations of depression. The results of previous comparative studies showed that the proportion of *no motion* was significantly higher in depressed patients than in the normal group (34, 55), but no significant difference was found in the comparison of individuals with schizophrenia and the normal group (27). The distorted characteristics represent a state of stress, and a *leaning house* suggested great stress in subjects. It was significantly reflected in individuals with both depression and anxiety disorders, appearing much more frequently in these groups than in the normal group (38, 56). Furthermore, meticulous drawings have been shown to represent sensitivity and irritability, coinciding with the clinical manifestations of anxiety disorders (15). Thus, a *decorated roof* was more frequently observed in the drawings of patients with affective-type disorders (57). Based on these findings, attention should be focused on distortion and excessive details in drawings when screening for affective-type disorders (e.g., depression and anxiety).

In addition, thought-specific indicators included *excessive separation among items*, *no window*, *loss of facial features*, and *inappropriate body proportions*. *Excessive separation among items* means that the house, tree, and person are separate and independent, which is more consistent with the broken and detached thinking of patients with thought-type disorders (e.g., schizophrenia). The results of the comparison study showed that this characteristic was only present in the schizophrenia group and not in the normal group (41, 58). However, no significant difference was found in the anxiety disorder group compared to the normal group (15). In addition, a comparison study found that 32.7% of patients with schizophrenia did not draw windows, and another 8.2% drew cutoff or odd windows, while 91.8% of the normal group drew regular windows (27). Relative to the normal group, *no window* is more likely to appear in the drawings of patients with schizophrenia, and there is a significant increase after treatment (37). However, there was no significant difference between the depressed and normal groups (50, 59). In addition, *loss of facial features* and *inappropriate body proportions* are more common due to the wild imaginations of thought-category disorder patients (60). Some patients may experience physical discomfort that is projected into their drawings. Many previous studies support this result, and found that schizophrenic patients were more likely to draw people with disproportionate head-to-body ratios, but no significant differences were found in another comparative study of depressed and normal individuals (27, 56, 61). Based on these findings, when screening for and diagnosing thought-type disorders (e.g., schizophrenia), focus should be placed on the obvious absence or excessive separation of drawing characteristics.

Common indicators of mental disorders included *no additional decoration*, *simplified drawings*, *very small houses*, *two-dimensional houses*, and *very small trees*. *Simplified drawings* without additional decoration have been proven to be significant predictors of mental disorders, implying that subjects are unresponsive and lack enthusiasm and motivation for life, which are typical symptoms of mental disorders. Many previous comparative studies on mental disorders such as depression and schizophrenia and normal individuals have found significant differences (19, 62). As mentioned previously, *very small or two-dimensional houses* and *very small trees* reflect the low psychological energy and insecurity of the subjects, and all appeared much more frequently in patients with depression, anxiety, and schizophrenia than in the normal group (34, 39, 41). Therefore, the common characteristics of mental disorders all reflect the lack of mental motivation. Based on the above findings, oversimplified painting, small drawing size, and small imagery should be of concern regardless of which mental disorders are being screened for and diagnosed.

### Strengths and limitations

This study is innovative in some ways. First, this paper innovatively integrates the characteristics of drawing in related studies since the development and application of HTP measurement through meta-analysis. This provides a reference standard for the selection of indicators in future HTP studies and offers the possibility for the development of objectification of the test (10, 12). In future studies, objectified indicators should be selected, and feature coding criteria should be formed to continuously promote the formation of an objectified HTP system. Second, this study found indicators specific to thought-type disorders and indicators specific to affective-type disorders and explored the theoretical implications, thus forming a theoretical guide for HTP testing. In the future, we should explore the predictive indicators of drawing for different psychological traits or mental disorders and continuously improve the theoretical guidance and application value of the HTP test. Finally, the predictive characteristics derived from this study can provide a basis for the screening and diagnosis of mental disorders, and their use in combination with the scale can improve the accuracy of mental disorder diagnosis (21). Meanwhile, the validity of the drawing characteristics of the HTP test needs to be continuously verified in clinical practice, which will in turn form an objective, complete, and valid predictor of mental disorders.

This study also has some shortcomings. First, it is still unclear whether subjects from other regions would have similar results because the included study population mainly originated from Asia, and only Chinese and English databases were searched. Second, it is difficult to classify drawing characteristics completely independent of each other when we encode them, resulting in overlapping meanings of certain characteristics. For example, *shaded or blackened drawings* contained *shaded or blackened persons*, while *incomplete persons* contained *complete or partial loss of limbs*. Attention should be given to the selection and interpretation of such drawing characteristics. Third, some of the drawing characteristics have been studied less often, which may have some influence on the accuracy of the results. More caution should be exercised, and more verification should be performed in interpreting these characteristics. Fourth, limited by the lack of basic information reported in the current literature, this study only explored the classification of mental disorders and could not explore the differences in gender and age. The analysis of the causes of drawing characteristics needs further depth. Finally, subgroup analysis can only explore two categories of mental disorders, and it is difficult to achieve a more refined classification, such as depression and anxiety, in affective-type disorders. It can be speculated that drawings with *missing or very small person* is more likely associated with depression, while *decorated roofs* are more consistent with anxiety. These results need to be further tested in future studies.

## Conclusion

In this study, we found that the greatest predictor of mental disorders was the whole drawing characteristic, followed in order by house, tree, and person characteristics. The drawing characteristics that significantly predicted mental disorders can be grouped into the following four categories: item absence, bizarre or distortion, excessive details, and small or simplified. Moreover, subgroup analysis distinguished between affective-specific indicators, thought-specific indicators, and common indicators of mental disorders. The above findings can provide reference standards for the selection of drawing characteristics and provide theoretical guidance for the screening and clinical diagnosis of mental disorders.

## Data availability statement

The original contributions presented in this study are included in the article/Supplementary material, further inquiries can be directed to the corresponding author.

## Author contributions

HG and TC contributed to the conception and design of the study. BF organized the database and performed the statistical analysis. HG, TC, and BF wrote the draft of the manuscript. TC, YM, XZ, HF, and ZD reviewed and edited the manuscript. TC and QG supervised the study and acquired funding. All authors contributed to the manuscript revision and read and approved the submitted version.
