# Supplementary material for: Analysis of the screening and predicting characteristics of the house-tree-person drawing test for mental disorders: A systematic review and meta-analysis
Source: Front Psychiatry. 2023 Jan 4;13:1041770. doi: 10.3389/fpsyt.2022.1041770 (PMC9848786; doi:10.3389/fpsyt.2022.1041770)
Supplement: Supplementary file 1 [file Table_1.docx]

Analysis of the Screening and Predicting Characteristics of the House-Tree-Person Drawing Test for Mental Disorders: A Systematic Review and Meta-analysis

Guo et al.

Supplementary Material

# Supplementary Tables

Table S1 Search strategy for Pubmed

| Search | Query |
| --- | --- |
| #1 | ("home environment"[MeSH Terms] OR ("home"[All Fields] AND "environment"[All Fields]) OR "home environment"[All Fields] OR "house"[All Fields] OR "houses"[All Fields] OR "housing"[MeSH Terms] OR "housing"[All Fields] OR "housed"[All Fields] OR "housings"[All Fields]) AND ("trees"[MeSH Terms] OR "trees"[All Fields] OR "tree"[All Fields]) AND ("person s"[All Fields] OR "personable"[All Fields] OR "personableness"[All Fields] OR "personal"[All Fields] OR "personalisation"[All Fields] OR "personalise"[All Fields] OR "personalised"[All Fields] OR "personalising"[All Fields] OR "personality"[MeSH Terms] OR "personality"[All Fields] OR "personalities"[All Fields] OR "personality s"[All Fields] OR "personalization"[All Fields] OR "personalize"[All Fields] OR "personalized"[All Fields] OR "personalizes"[All Fields] OR "personalizing"[All Fields] OR "personally"[All Fields] OR "personals"[All Fields] OR "persons"[MeSH Terms] OR "persons"[All Fields] OR "person"[All Fields]) |
| #2 | ("drawing"[All Fields] OR "drawings"[All Fields] OR "draws"[All Fields]) AND ("research design"[MeSH Terms] OR ("research"[All Fields] AND "design"[All Fields]) OR "research design"[All Fields] OR "test"[All Fields]) |
| #3 | "projective techniques"[MeSH Terms] OR ("projective"[All Fields] AND "techniques"[All Fields]) OR "projective techniques"[All Fields] OR ("projective"[All Fields] AND "test"[All Fields]) OR "projective test"[All Fields] |
| #4 | "K-HTP"[All Fields] OR "S-HTP"[All Fields] |
| #5 | "HTP"[All Fields] AND ("research design"[MeSH Terms] OR ("research"[All Fields] AND "design"[All Fields]) OR "research design"[All Fields] OR "test"[All Fields]) |
| #6 | "House-Tree-Person"[All Fields] |
| #7 | #1 OR #2 OR #3 OR #4 OR #5 OR #6 |
|  |  |
|  |  |
|  |  |
|  |  |

Table S2 Search strategy for EMBASE

| Search | Query |
| --- | --- |
| #1 | ('home environment':ab,ti or ('home':ab,ti and 'environment':ab,ti ) or 'home environment':ab,ti or 'house':ab,ti or 'houses':ab,ti or 'housing':ab,ti or 'housing':ab,ti or 'housed':ab,ti or 'housings':ab,ti ) and ('trees':ab,ti or 'trees':ab,ti or 'tree':ab,ti ) and ('person s':ab,ti or 'personable':ab,ti or 'personableness':ab,ti or 'personal':ab,ti or 'personalisation':ab,ti or 'personalise':ab,ti or 'personalised':ab,ti or 'personalising':ab,ti or 'personality':ab,ti or 'personality':ab,ti or 'personalities':ab,ti or 'personality s':ab,ti or 'personalization':ab,ti or 'personalize':ab,ti or 'personalized':ab,ti or 'personalizes':ab,ti or 'personalizing':ab,ti or 'personally':ab,ti or 'personals':ab,ti or 'persons':ab,ti or 'persons':ab,ti or 'person':ab,ti ) |
| #2 | ('drawing':ab,ti or 'drawings':ab,ti or 'draws':ab,ti) and ('research design':ab,ti or ('research':ab,ti and 'design':ab,ti) or 'research design':ab,ti or 'test':ab,ti) |
| #3 | 'projective techniques':ab,ti or ('projective':ab,ti and 'techniques':ab,ti) or 'projective techniques':ab,ti or ('projective':ab,ti and 'test':ab,ti) or 'projective test':ab,ti |
| #4 | 'K-HTP':ab,ti or 'S-HTP':ab,ti |
| #5 | 'HTP':ab,ti and ('research design':ab,ti or ('research':ab,ti and 'design':ab,ti) or 'research design':ab,ti or 'test':ab,ti) |
| #6 | 'House-Tree-Person':ab,ti |
| #7 | #1 OR #2 OR #3 OR #4 OR #5 OR #6 |

Table S3 Search strategy for EBSCO

| Search | Query |
| --- | --- |
| #1 | (TX home environment OR (TX home AND TX environment) OR TX home environment OR TX house OR TX houses OR TX housing OR TX housing OR TX housed OR TX housings) AND (TX trees OR TX trees OR TX tree) AND (TX person s OR TX personable OR TX personableness OR TX personal OR TX personalisation OR TX personalise OR TX personalised OR TX personalising OR TX personality OR TX personality OR TX personalities OR TX personality s OR TX personalization OR TX personalize OR TX personalized OR TX personalizes OR TX personalizing OR TX personally OR TX personals OR TX persons OR TX persons OR TX person) |
| #2 | (TX drawing OR TX drawings OR TX draws) AND (TX research design OR (TX research AND TX design) OR TX research design OR TX test) |
| #3 | TX projective techniques OR (TX projective AND TX techniques) OR TX projective techniques OR (TX projective AND TX test) OR TX projective test |
| #4 | TX K-HTP OR TX S-HTP |
| #5 | TX HTP AND (TX research design OR (TX research AND TX design) OR TX research design OR TX test) |
| #6 | TX House-Tree-Person |
| #7 | #1 OR #2 OR #3 OR #4 OR #5 OR #6 |

Table S4 Search strategy for Web of Science

| Search | Query |
| --- | --- |
| #1 | (TS=("home environment") OR (TS=("home") AND TS=("environment")) OR TS=("home environment") OR TS=("house") OR TS=("houses") OR TS=("housing") OR TS=("housing") OR TS=("housed") OR TS=("housings")) AND ("trees") OR "trees") OR "tree")) AND (TS=("person s") OR TS=("personable") OR TS=("personableness") OR TS=("personal") OR TS=("personalisation") OR TS=("personalise") OR TS=("personalised") OR TS=("personalising") OR TS=("personality") OR TS=("personality") OR TS=("personalities") OR TS=("personality s") OR TS=("personalization") OR TS=("personalize") OR TS=("personalized") OR TS=("personalizes") OR TS=("personalizing") OR TS=("personally") OR TS=("personals") OR TS=("persons") OR TS=("persons") OR TS=("person")) |
| #2 | (TS=("drawing") OR TS=("drawings") OR TS=("draws")) AND (TS=("research design") OR (TS=("research") AND TS=("design")) OR TS=("research design") OR TS=("test")) |
| #3 | TS=("projective techniques") OR (TS=("projective") AND TS=("techniques")) OR TS=("projective techniques") OR (TS=("projective") AND TS=("test")) OR TS=("projective test") |
| #4 | TS=("K-HTP") OR TS=("S-HTP") |
| #5 | TS=("HTP") AND (TS=("research design") OR (TS=("research") AND TS=("design")) OR TS=("research design") OR TS=("test")) |
| #6 | TS=("House-Tree-Person") |
| #7 | #1 OR #2 OR #3 OR #4 OR #5 OR #6 |

Table S5 Search strategy for CNKI, VIP and Wanfang databases

| Search | Query |
| --- | --- |
| #1 | 房树人绘画测验[主题] |
| #2 | 房树人测验[主题] |
| #3 | 房树人[主题] |
| #4 | HTP[主题] |
| #5 | S-HTP[主题] |
| #6 | K-HTP[主题] |
| #7 | #1-#6/OR |
| #8 | 投射测验[主题] |
| #9 | 绘画测验[主题] |
| #10 | #8-#10/OR |
| #11 | #7 AND #11 |

| **Section and Topic** | **Item #** | **Checklist item** | **Location where item is reported** |
| --- | --- | --- | --- |
| **TITLE** | | |  |
| Title | 1 | Identify the report as a systematic review. | Title |
| **ABSTRACT** | | |  |
| Abstract | 2 | See the PRISMA 2020 for Abstracts checklist. | Abstract |
| **INTRODUCTION** | | |  |
| Rationale | 3 | Describe the rationale for the review in the context of existing knowledge. | Introduction |
| Objectives | 4 | Provide an explicit statement of the objective(s) or question(s) the review addresses. | Introduction |
| **METHODS** | | |  |
| Eligibility criteria | 5 | Specify the inclusion and exclusion criteria for the review and how studies were grouped for the syntheses. | Methods, Inclusion and exclusion criteria |
| Information sources | 6 | Specify all databases, registers, websites, organisations, reference lists and other sources searched or consulted to identify studies. Specify the date when each source was last searched or consulted. | Methods, Search Strategy |
| Search strategy | 7 | Present the full search strategies for all databases, registers and websites, including any filters and limits used. | Supplementary material, Table S1, S2, S3, S4, S5, S6, S7 |
| Selection process | 8 | Specify the methods used to decide whether a study met the inclusion criteria of the review, including how many reviewers screened each record and each report retrieved, whether they worked independently, and if applicable, details of automation tools used in the process. | Methods, Inclusion and exclusion criteria |
| Data collection process | 9 | Specify the methods used to collect data from reports, including how many reviewers collected data from each report, whether they worked independently, any processes for obtaining or confirming data from study investigators, and if applicable, details of automation tools used in the process. | Methods, Coding Procedures |
| Data items | 10a | List and define all outcomes for which data were sought. Specify whether all results that were compatible with each outcome domain in each study were sought (e.g. for all measures, time points, analyses), and if not, the methods used to decide which results to collect. | Methods, Coding Procedures |
|  | 10b | List and define all other variables for which data were sought (e.g. participant and intervention characteristics, funding sources). Describe any assumptions made about any missing or unclear information. | Methods, Inclusion and exclusion criteria |
| Study risk of bias assessment | 11 | Specify the methods used to assess risk of bias in the included studies, including details of the tool(s) used, how many reviewers assessed each study and whether they worked independently, and if applicable, details of automation tools used in the process. | Methods, Quality Assessment, Publication Bias |
| Effect measures | 12 | Specify for each outcome the effect measure(s) (e.g. risk ratio, mean difference) used in the synthesis or presentation of results. | Methods, Statistical Analysis |
| Synthesis methods | 13a | Describe the processes used to decide which studies were eligible for each synthesis (e.g. tabulating the study intervention characteristics and comparing against the planned groups for each synthesis (item #5)). | Methods, Coding Procedures |
|  | 13b | Describe any methods required to prepare the data for presentation or synthesis, such as handling of missing summary statistics, or data conversions. | Methods, Coding Procedures |
|  | 13c | Describe any methods used to tabulate or visually display results of individual studies and syntheses. | Methods, Statistical Analysis |
|  | 13d | Describe any methods used to synthesize results and provide a rationale for the choice(s). If meta-analysis was performed, describe the model(s), method(s) to identify the presence and extent of statistical heterogeneity, and software package(s) used. | Methods, Statistical Analysis |
|  | 13e | Describe any methods used to explore possible causes of heterogeneity among study results (e.g. subgroup analysis, meta-regression). | Methods, Statistical Analysis |
|  | 13f | Describe any sensitivity analyses conducted to assess robustness of the synthesized results. | None |
| Reporting bias assessment | 14 | Describe any methods used to assess risk of bias due to missing results in a synthesis (arising from reporting biases). | Methods, Publication Bias |
| Certainty assessment | 15 | Describe any methods used to assess certainty (or confidence) in the body of evidence for an outcome. | Methods, Publication Bias |
| **RESULTS** | | |  |
| Study selection | 16a | Describe the results of the search and selection process, from the number of records identified in the search to the number of studies included in the review, ideally using a flow diagram. | Results, Study selection and Characteristics, Figure 1 |
|  | 16b | Cite studies that might appear to meet the inclusion criteria, but which were excluded, and explain why they were excluded. | Figure 1 |
| Study characteristics | 17 | Cite each included study and present its characteristics. | Results, Study selection and Characteristics |
| Risk of bias in studies | 18 | Present assessments of risk of bias for each included study. | Results, Analysis of Publication Bias |
| Results of individual studies | 19 | For all outcomes, present, for each study: (a) summary statistics for each group (where appropriate) and (b) an effect estimate and its precision (e.g. confidence/credible interval), ideally using structured tables or plots. | None |
| Results of syntheses | 20a | For each synthesis, briefly summarise the characteristics and risk of bias among contributing studies. | Results, Predictive Effect of Mental Disorders, Analysis of Publication Bias |
|  | 20b | Present results of all statistical syntheses conducted. If meta-analysis was done, present for each the summary estimate and its precision (e.g. confidence/credible interval) and measures of statistical heterogeneity. If comparing groups, describe the direction of the effect. | Results, Table 2 |
|  | 20c | Present results of all investigations of possible causes of heterogeneity among study results. | Results, Subgroup Analysis, Table 3 |
|  | 20d | Present results of all sensitivity analyses conducted to assess the robustness of the synthesized results. | None |
| Reporting biases | 21 | Present assessments of risk of bias due to missing results (arising from reporting biases) for each synthesis assessed. | Results, Analysis of Publication Bias |
| Certainty of evidence | 22 | Present assessments of certainty (or confidence) in the body of evidence for each outcome assessed. | None |
| **DISCUSSION** | | |  |
| Discussion | 23a | Provide a general interpretation of the results in the context of other evidence. | Discussion, Drawing characteristics of HTP |
|  | 23b | Discuss any limitations of the evidence included in the review. | Discussion, Strengths and Limitations |
|  | 23c | Discuss any limitations of the review processes used. | Discussion, Strengths and Limitations |
|  | 23d | Discuss implications of the results for practice, policy, and future research. | Discussion, Strengths and Limitations |
| **OTHER INFORMATION** | | |  |
| Registration and protocol | 24a | Provide registration information for the review, including register name and registration number, or state that the review was not registered. | None |
|  | 24b | Indicate where the review protocol can be accessed, or state that a protocol was not prepared. | None |
|  | 24c | Describe and explain any amendments to information provided at registration or in the protocol. | None |
| Support | 25 | Describe sources of financial or non-financial support for the review, and the role of the funders or sponsors in the review. | Funding |
| Competing interests | 26 | Declare any competing interests of review authors. | Conflicts of Interest |
| Availability of data, code and other materials | 27 | Report which of the following are publicly available and where they can be found: template data collection forms; data extracted from included studies; data used for all analyses; analytic code; any other materials used in the review. | None |

*From:*  Page MJ, McKenzie JE, Bossuyt PM, Boutron I, Hoffmann TC, Mulrow CD, et al. The PRISMA 2020 statement: an updated guideline for reporting systematic reviews. BMJ 2021;372:n71. doi: 10.1136/bmj.n71

For more information, visit: <http://www.prisma-statement.org/>
